# The Role of Melatonin on Behavioral Changes and Concomitant Oxidative Stress in icvAβ_1-42_ Rat Model with Pinealectomy

**DOI:** 10.3390/ijms222312763

**Published:** 2021-11-25

**Authors:** Rumiana Tzoneva, Irina Georgieva, Natasha Ivanova, Veselina Uzunova, Zlatina Nenchovska, Sonia Apostolova, Tzveta Stoyanova, Jana Tchekalarova

**Affiliations:** 1 Institute of Biophysics and Biomedical Engineering, Bulgarian Academy of Sciences, Acad. G. Bonchev Street, Block 21, 1113 Sofia, Bulgaria; georgieva.irina5@gmail.com (I.G.); vesi.uzunova@abv.bg (V.U.); sonia_apostolova@yahoo.com (S.A.); 2 Institute of Neurobiology, Bulgarian Academy of Sciences, Acad. G. Bonchev Street, Block 23, 1113 Sofia, Bulgaria; ivanova_nm@yahoo.com (N.I.); zuzania@abv.bg (Z.N.); tzafti@abv.bg (T.S.)

**Keywords:** icvAβ_1-42_, pinealectomy, melatonin, anxiety, memory, oxidative stress, rat

## Abstract

One of the pathological hallmarks of Alzheimer’s disease (AD) associated with its progression that contributes to β-amyloid (Aβ) generation is oxidative stress (OS). Clinical data suggest that melatonin is a potent antioxidant that might be effective in the adjunctive therapy of this neurodegenerative disease. The present study aimed to explore the role of melatonin on behavioral changes and markers of OS in three rat models, namely, pinealectomy (pin) model of melatonin deficit, intracerebroventricular (icv)Aβ_1-42_ model of AD, and combination of both pin and Aβ_1-42_ model (pin+icvAβ_1-42_). The chronic injection with vehicle/melatonin (50 mg/kg, i.p. for 40 days) started on the same day of sham/pin and icv vehicle/Aβ_1-42_ infusion procedures. Anxiety in the open field and the elevated plus-maze test and cognitive responses in the object recognition test were tested between the 30th–35th day after the surgical procedures. Markers of OS in the frontal cortex (FC) and hippocampus were detected by the ELISA method. Melatonin treatment corrected the exacerbated anxiety response only in the pin+icvAβ_1-42_ model while it alleviated the cognitive impairment in the three models. Pinealectomy disturbed the antioxidant system via enhanced SOD activity and decreased GSH levels both in the FC and hippocampus. The Aβ_1-42_ model decreased the SOD activity in the FC and elevated the MDA level in the two brain structures. The pin+icvAβ_1-42_ model impaired the antioxidant system and elevated lipid peroxidation. Melatonin supplementation restored only the elevated MDA level of icvAβ_1-42_ and pin+icvAβ_1-42_ model in the hippocampus. In conclusion, our study reveals that the pin+icvAβ_1-42_ rat model triggers more pronounced anxiety and alterations in markers of OS that may be associated with melatonin deficit concomitant to icvAβ_1-42_-induced AD pathology.

## 1. Introduction

The main pathological characteristics of Alzheimer’s disease (AD) are the formations of extracellular Aβ plaques and intraneuronal deposits of neurofibrillary tangles (NFTs). Oxidative stress (OS) is an important pathogenic mechanism of AD which appears as a prominent and early feature of this neurodegenerative disease and leads to the formation of free radicals and subsequent damage of specific brain regions, including the frontal cortex (FC) and hippocampus [1,2,3,4,5]. Oxidative stress is an imbalance between the reactive oxygen species (ROS) and the antioxidant system that removes them. This state may be due to overproduction of ROS or a reduction in the antioxidant defense system, which has a high oxidative capacity while its ability to fight the OS is limited [6,7]. Brain tissue has multiple potential sources of ROS [8,9]. One of the most relevant hypotheses describing OS as the main factor in AD pathophysiology is because neuronal cells have a higher intake of oxygen, elevated lipid content, and a lower content of antioxidant enzymes such as Cu/Zn-superoxide dismutase (SOD), glutathione (GSH) and catalase compared to other types of cells, making them more vulnerable to changes in OS [10,11,12,13]. Together with the presence of Aβ and NFT, these factors lead to mitochondrial dysfunction and neuronal cell death [14]. Therefore, one of the promising therapies of AD is the reduction of OS through antioxidant treatment [15,16,17].

Melatonin (N-acetyl-5-methoxytryptamine) is a crucial endogenous indoleamine secreted by the pineal gland and other extrapineal tissues such as the gastrointestinal tract [18]. In addition to its critical role in regulating circadian rhythms, melatonin and its metabolites have well-known antioxidant properties in the central nervous system (CNS) [19,20]. Furthermore, along with the antioxidant activity of melatonin to CNS, there is evidence that suggests the efficacy of melatonin in preventing oxidative stress in cells as erythrocytes in a concentration-related manner [21]. Clinical evidence suggests that the diminished function of this hormone represents an earlier biomarker of AD [22,23,24,25]. Changes in melatonin (MT) receptors associated with this melatonin deficit have also been reported in patients with AD [26,27]. Melatonin can quickly cross the blood-brain barrier, making the exogenous melatonin administration a drug of choice against circadian desynchronization, emotional disturbance, and memory decline in patients with AD [28,29,30,31,32].

Pinealectomy was established as an experimentally induced melatonin-deficient animal model to evaluate the occurrence of scoliosis in rodents [33] and appears to be a good model to study melatonin deficiency and pathway dysfunction in the pathogenesis of neurodegenerative diseases like multiple sclerosis, AD, and Parkinson’s disease [34]. Most AD models were performed using synthetic Aβ, Aβ_1–40_, or Aβ_1-42_ analogous to peptides found in neuritic plaques in AD patients [35]. The central administration of Aβ induced learning, and memory deficits in rats [36], cholinergic dysfunction [37], neuronal apoptosis [38], OS [39], and neuroinflammation [40]. Specifically, microinjections of Aβ_1-42_ impaired memory in various memory tasks, including Y-maze, radial arm-maze, Morris water-maze tests, and passive avoidance test [41,42,43].

In the present study, we aimed to introduce a new AD animal model concomitant with melatonin deficiency obtained by a combination of pinealectomy and icv infusion of Aβ_1-42_ (pin+icvAβ_1-42_), to check whether: (i) melatonin deficit can exacerbate the expected icvAβ_1-42_-related alterations in behavior and markers of OS and (ii) melatonin supplementation can correct them in a rat pin+icvAβ_1-42_model.

## 2. Results

### 2.1. Effects of Pinealectomy and Melatonin Treatment on Behavioral Responses in icvAβ Rat Model

#### 2.1.1. Open Field Test

**Figure 1:**
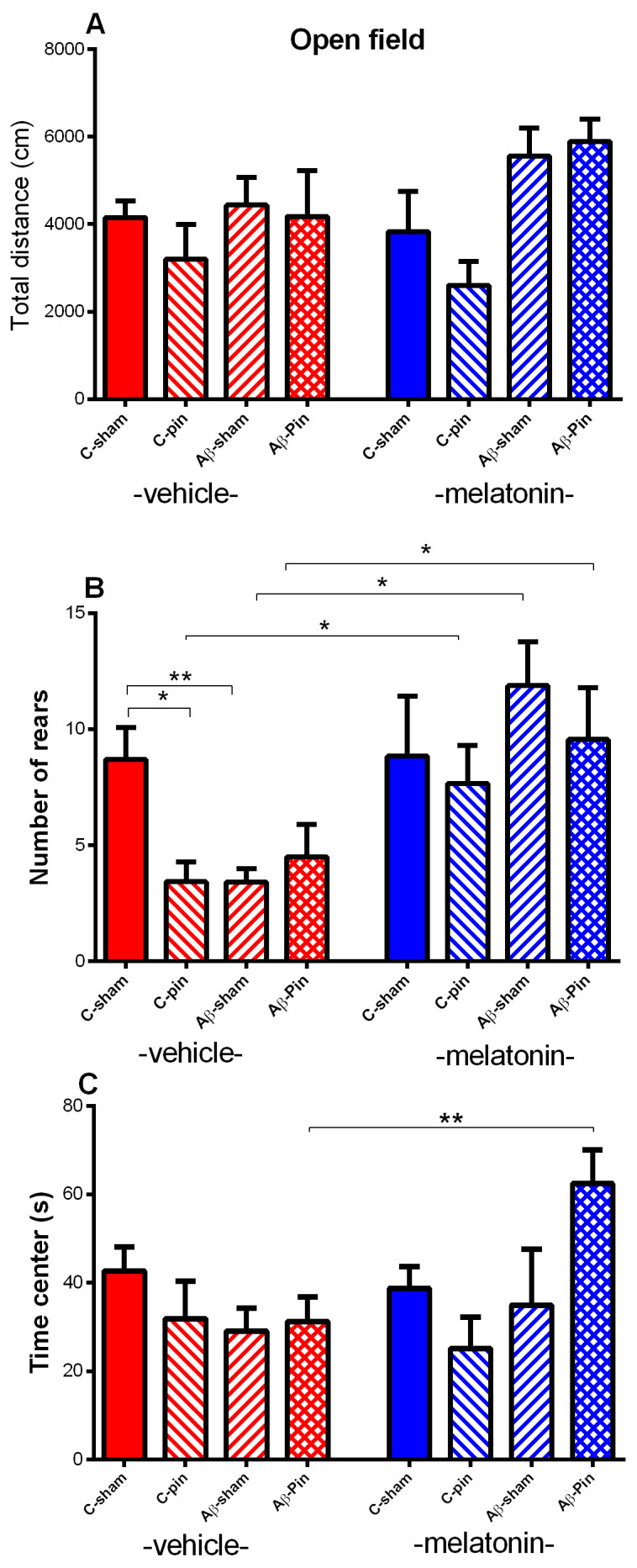
The influence of pinealectomy (pin) and chronic treatment with melatonin (mel) on icvAβ_1-42_ peptide-related effect on total distance travelled (cm) (**A**), number of rears (**B**) and time (sec) spent in the center (**C**) in the open field test. Data are presented as mean ± S.E.M. * *p* = 0.0273 C-pin-veh compared to C-sham-veh group; * *p* = 0.014 Aβ-sham-veh compared to the C-sham-veh group, * *p* = 0.034 C-pin-mel group compared to C-pin-veh group; * *p* = 0.012 Aβ-sham-mel group compared to Aβ-sham-veh group; * *p* = 0.045 Aβ-pin-mel group compared to Aβ-pin-veh group (**B**); ** *p* = 0.009 Aβ-pin-mel compared to Aβ-pin-veh group (**C**).

The total motor activity was not affected by the three factors, Pinealectomy, Aβ, and Drug (*p* > 0.05) (Figure 1A). Changes in the vertical activity (rears) and time spent in the center is the behavioral marker of the altered anxiety response. The main effect of Pinealectomy [F_1,63_ = 6.039, *p* = 0.018], Aβ [F_1,63_ = 4.226, *p* = 0.043] and Drug was found for rears [F_1,63_ = 14.220, *p* < 0.001]. *Post hoc* test demonstrated that both, removal of the pineal gland, and addition of Aβ, diminished exploratory behavior (rears) in vehicle-treated groups compared to the C-sham-veh group (*p* = 0.0273; *p* = 0.014). The combination of both models showed identical to their individual effects (*p* > 0.05 Aβ-pin-veh group compared to C-pin-veh group and Aβ-sham-veh group). Melatonin treatment restored decreased vertical activity associated with pinealectomy (*p* = 0.034 C-pin-mel group compared to C-pin-veh group), Aβ accumulation (*p* = 0.012 Aβ-sham-mel group compared to Aβ-sham-veh group) and pin+Aβ treatment (*p* = 0.045 Aβ-pin-mel group compared to Aβ-pin-veh group) (Figure 1B). A significant Pinealectomy x Aβ x Drug interaction [F_1,63_ = 4.718, *p* = 0.035] was detected for the time spent in the aversive zone (center). *Post hoc* test confirmed that melatonin provoked an anxiolytic response in the Aβ-group with pinealectomy (*p* = 0.009 compared to Aβ-pin-veh group) (Figure 1C).

#### 2.1.2. Elevated plus Maze Test

**Figure 2:**
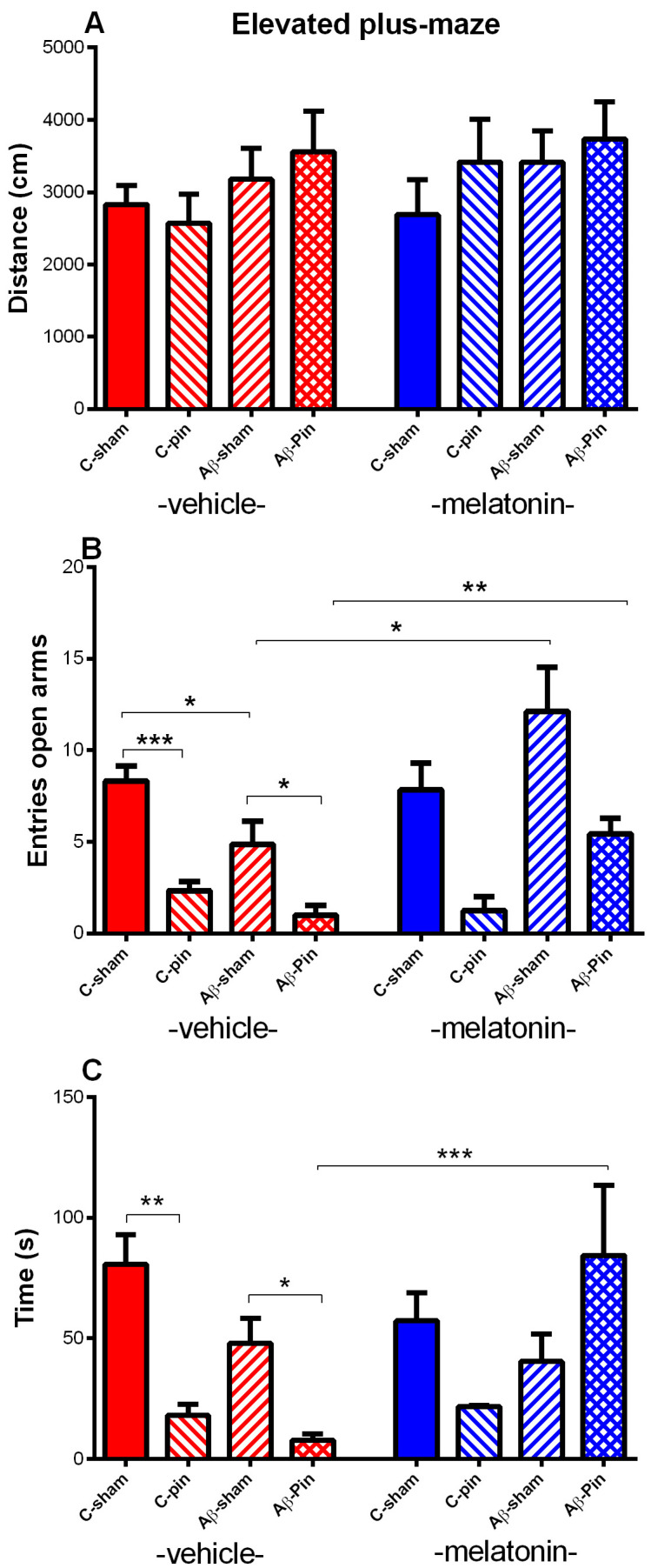
The influence of pin and chronic treatment with mel on icvAβ_1-42_ peptide-related effect on total distance travelled (cm) (**A**), number of entries in open arms (**B**) and time (sec) spent in the open arms (**C**) in the elevated plus-maze test. Data are presented as mean ± S.E.M. *** *p* < 0.001 C-pin-veh compared to C-sham-veh, * *p* = 0.026 Aβ-sham-veh compared to C-sham-veh * *p* = 0.035 Aβ-pin-veh compared to Aβ-sham-veh, * *p* = 0.014 Aβ-sham-mel compared to Aβ-sham-veh, ** *p* = 0.001 Aβ-pin-mel compared to Aβ-pin-veh (**B**); ** *p* = 0.006 C-pin-veh compared to C-sham-veh group, * *p* = 0.014 Aβ-pin-veh compared to Aβ-sham-veh group, *** *p* < 0.001 Aβ-pin-mel compared to Aβ-pin-veh group (**C**).

Like in the OF test, melatonin deficit, Aβ accumulation as well as melatonin treatment did not influence the total distance traveled (*p* > 0.05) (Figure 2A). The frequency of entries and time spent in the open arms of the EPM test is used to evaluate anxiety response. Main effect of Pinealectomy [F_1,63_ = 26.449, *p* < 0.001], Aβ [F_1,63_ = 5.134, *p* = 0.032] and Drug [F_1,58_ = 5.889, *p* = 0.019] with Aβ × Drug interaction [F_1,63_ = 8.796, *p* = 0.05] were observed for the number of entries in the open arms. The *post hoc* test showed that both melatonin deficit and Aβ accumulation significantly diminished the number of entries in the open arms (*p* < 0.001 C-pin-veh compared to C-sham-veh) (*p* = 0.026 Aβ-sham-veh compared to C-sham-veh) (Figure 2B). The melatonin deficit in a condition of Aβ accumulation additionally attenuated the number of entries in the aversive zone of the EPM (*p* = 0.035 Aβ-pin-veh compared to Aβ-sham-veh). The melatonin treatment restored Aβ-induced changes in anxiety response in both sham and group with pinealectomy (*p* = 0.014 and *p* = 0.001, respectively). As for the number of entries, main effects of Pinealectomy [F_1,63_= 4.112, *p* = 0.048] with Aβ × Pinealectomy [F_1,63_ = 4.885, *p* = 0.032] and Aβ × Drug [F_1,63_ = 5.862, *p* = 0.019] interaction were detected for the time spent in the open arms. The *post hoc* test demonstrated that removing the pineal gland decreased the time spent in the open arms (*p* = 0.006) (Figure 2C). While the icv. Aβ infusion in the Aβ-sham-veh group showed only a tendency to diminish the time spent in the open arms (*p* > 0.005), the removal of the pineal gland significantly increased the anxiety level in the Aβ-veh group (*p* = 0.014 Aβ-pin-veh compared to Aβ-sham-veh group). The melatonin treatment restored to control level anxiety response in the Aβ group with melatonin deficit (*p* < 0.001 Aβ-pin-mel compared to Aβ-pin-veh group).

#### 2.1.3. Object Recognition Test

**Figure 3:**
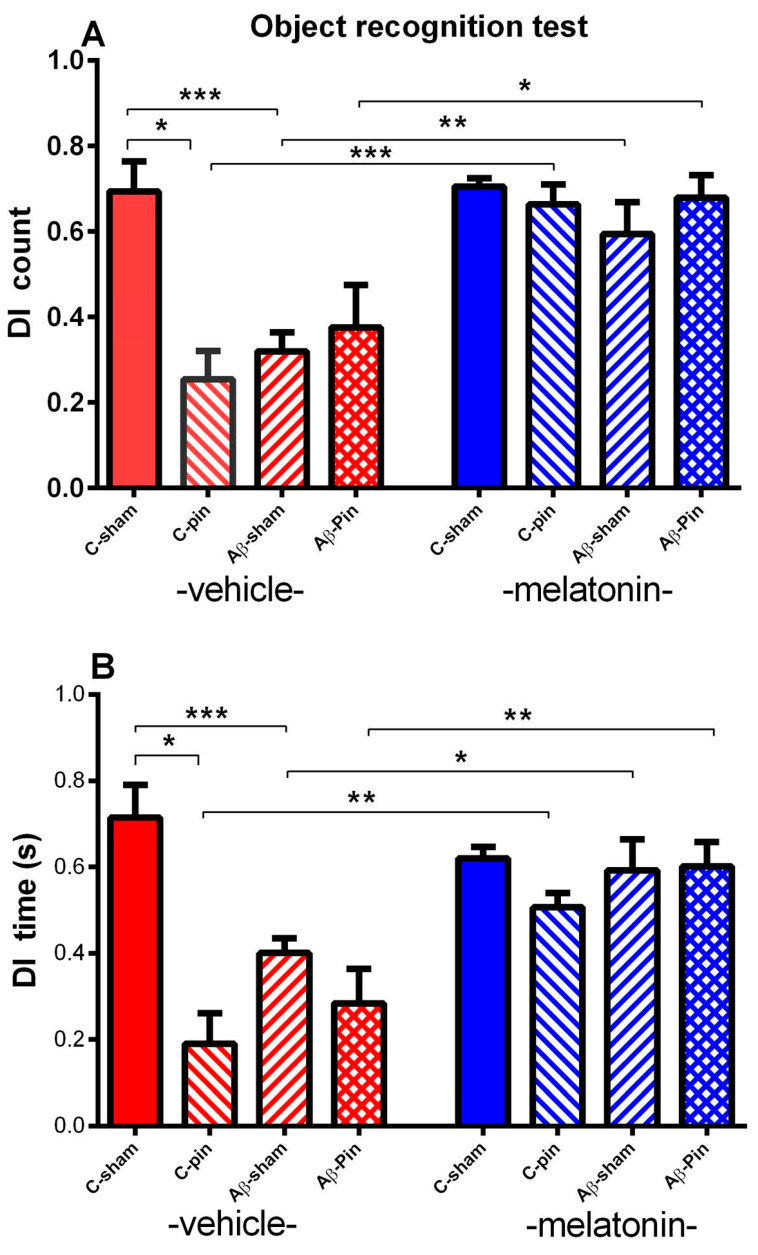
The influence of pin and chronic treatment with mel on discrimination index (DI) (count) (**A**) and DI (time) (sec) (**B**) in Object recognition test. Data are presented as mean ± S.E.M. * *p* = 0.037 C-pin-veh group compared to C–sham-veh group, *** *p* = 0.0007 Aβ-sham-veh group compared to C–sham-veh group, *** *p* < 0.001 C-pin-mel group compared to C-pin-veh group, * *p* = 0.006 Aβ-sham-mel compared to Aβ-sham–veh and * *p* = 0.013 Aβ-pin-mel compared to Aβ-pin–veh (**A**); * *p* = 0.012 C-pin-veh group compared to C–sham-veh group; *** *p* = 0.001 Aβ-sham-veh group compared to C-sham-veh group, ** *p* = 0.006 C-pin-mel group compared to C-pin-veh group, * *p* = 0.013 Aβ-sham-mel compared to Aβ-sham-veh, *p* = 0.009 Aβ-pin-mel compared to Aβ-pin–veh group.

Three-way ANOVA revealed the main effect of Pinealectomy [F_1,63_ = 5.022, *p* = 0.029], Aβ [F_1,63_ = 4.514, *p* = 0.038] and Drug [F_1,63_ = 15.419, *p* < 0.001] with Pinealectomy × Aβ × Drug interaction [F_1,63_ = 4.361, *p* = 0.045] for the DI of counts. The *post-hoc* test demonstrated that removal of the pineal gland as well as Aβ accumulation worsened object recognition memory (*p* = 0.037 C-pin-veh group compared to C–sham-veh group; *p* = 0.0007 Aβ-sham-veh group compared to C–sham-veh group) (Figure 3A). The chronic melatonin treatment managed to alleviate the negative impact of both pinealectomy and Aβ accumulation (*p* < 0.001 pin-sham-mel compared to pin-sham-veh; *p* = 0.006 Aβ-sham-mel compared to Aβ -sham-veh) as well as the effect of melatonin deficit in the Aβ group (*p* = 0.013 Aβ-pin-mel compared to Aβ-pin-veh).

The main effect of Pinealectomy [F_1,63_ = 7.777, *p* = 0.007], Aβ [F_1,63_ = 5.115, *p* = 0.028] and Drug [F_1,63_ = 7.399, *p* = 0.009] with Aβ × Drug interaction [F_1,63_ = 4.032, *p* = 0.05] and Pinealectomy × Aβ × Drug interaction [F_1,63_ = 3.032, *p* = 0.045] for the DI of time was demonstrated. As for DI of counts the *post-hoc* test showed that both melatonin deficit and Aβ accumulation, alone and in combination, impaired the object recognition memory (*p* = 0.012 pin-C-veh group compared to sham-C–veh group; *p* = 0.001 Aβ-sham-veh group compared to C–sham-veh group). Further, melatonin treatment restored recognition memory in the two models (pinealectomy and icvAβ_1-42_) as well as their combination (*p* = 0.006 pin-sham-mel compared to pin-sham-veh; *p* = 0.013 Aβ-sham-mel compared to Aβ-sham–veh; *p* = 0.009 Aβ-pin-mel compared to Aβ-pin–veh, respectively) (Figure 3B).

### 2.2. Effects of Pinealectomy and Melatonin Treatment on Markers of Oxidative Stress in the Frontal Cortex and Hippocampus in icvAβ Rat Model

#### 2.2.1. GSH Activity in the FC

**Figure 4:**
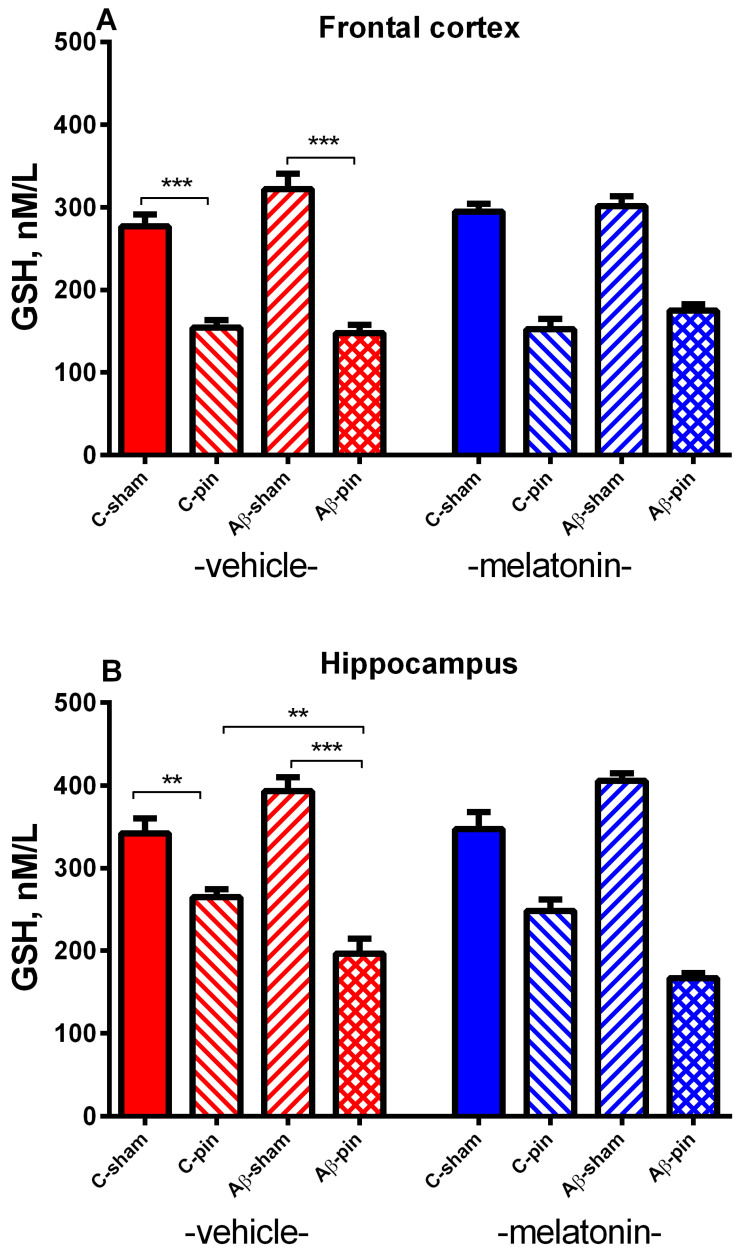
The influence of pinealectomy (pin) and chronic treatment with melatonin (mel) on GSH activity in the frontal cortex (**A**) and GSH activity in the hippocampus (**B**). Data are presented as mean ± S.E.M. *** *p* < 0.001, C-pin-veh group compared to C-sham-veh; Aβ -pin-veh group compared to Aβ-sham-veh group (**A**) ** *p* = 0.005, C-pin-veh group compared to C-sham-veh group; ** *p* = 0.0065 Aβ-pin-veh group compared to C-pin-veh group; *** *p* < 0.001 Aβ-pin-veh group compared to Aβ-sham-veh group (**B**).

A main effect of Pinealectomy [F_1,63_ = 234.065, *p* <0.001] for GSH levels was detected in the FC. The *post hoc* test demonstrated that removing the pineal gland induced a significant decrease of GSH levels in the FC (*p* < 0.001, C- pin-veh group compared to the C-sham-veh group; Aβ-pin-veh group compared to Aβ-sham-veh group). Melatonin did not affect the GSH levels in this brain structure *p* > 0.05) (Figure 4A).

#### 2.2.2. GSH Activity in the Hippocampus

**Figure 5:**
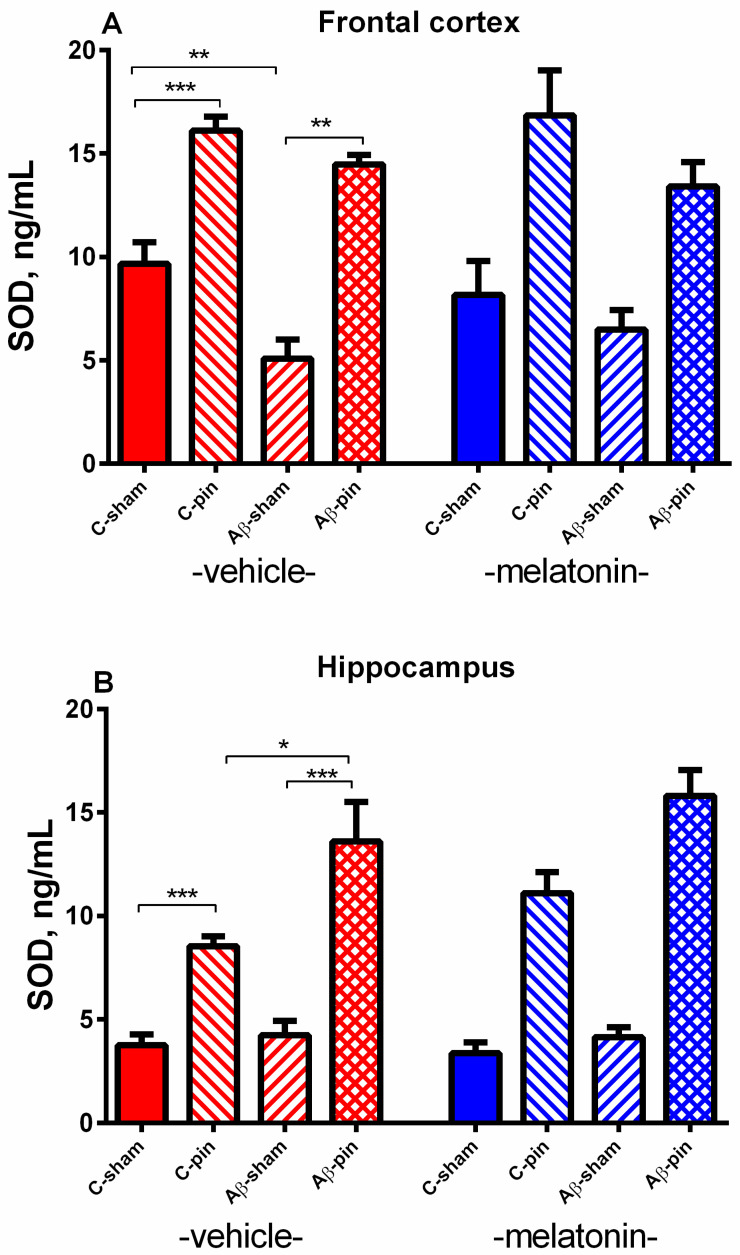
The influence of pinealectomy (pin) and chronic treatment with melatonin (mel) on SOD activity in the frontal cortex (**A**) and hippocampus (**B**). Data are presented as mean ± S.E.M. * *p* < 0.001 C-pin-veh group compared to C-sham-veh group; ** *p* < 0.0013, Aβ-sham-veh group compared to C-sham-veh group; ** *p* = 0.002 Aβ-pin-veh group compared to Aβ-sham-veh group (**A**) *** *p* < 0.001 C-pin-veh group compared to C-sham-veh group; * p = 0.05, Aβ -pin-veh group compared to sham-pin-veh; *** *p* = 0.0002, Aβ-pin-veh group compared to Aβ-sham-veh group (**B**).

Like in the FC, the main effect of Pinealectomy [F_1,63_ = 213.284, *p* < 0.001] with Pinealectomy × Aβ interaction [F_2,63_ = 16.852, *p* < 0.001] for the GSH levels was demonstrated after Aβ accumulation. The *post-hoc* test showed a statistically significant decrease of the GSH levels both in the C-pin and the Aβ rats with pinealectomy (*p* < 0.005, C-pin-veh group compared to C–sham-veh group; *p* = 0.0065, Aβ-pin-veh group compared to C-pin-veh group; *p* < 0.001, Aβ-pin-veh group compared to Aβ-C-veh group) (Figure 5B). Similar to the FC, melatonin did not affect GSH levels in the hippocampus.

#### 2.2.3. SOD Activity in the FC

Three-way ANOVA revealed a main Pinealectomy effect for the activity of SOD in the FC [F_1,63_ = 21.560, *p* < 0.001]. The *post-hoc* test demonstrated that the removal of the pineal gland increased the SOD activity in both the control and Aβ group (*p* < 0.001, C-pin-veh group compared to C-sham-veh group; *p* = 0.0002, Aβ-pin-veh group compared to Aβ-sham-veh group) (Figure 5A). In the opposite, a decreased SOD activity of rats icv infused with Aβ was detected in the FC (*p* < 0.0013, Aβ-sham-veh group compared to C-sham-veh group). As for the GSH level, melatonin did not affect SOD activity in the FC.

#### 2.2.4. SOD Activity in the Hippocampus

A main Pinealectomy effect [F_1,63_ = 82.450, *p* < 0.001] with Pinealectomy × Aβ [F_2,63_ = 5.144, *p* = 0.01] interaction was detected in the hippocampus. Like in the FC, the *post hoc* test showed that pinealectomy elevated the SOD activity in the sham group and more profoundly in the icvAβ-pin group (*p* = 0.0002, C-pin-veh group compared to C-sham-veh group; *p* = 0.05, Aβ-pin-veh group compared to sham-pin-veh; *p* = 0.0002 Aβ-pin-veh group compared to Aβ-sham-veh group) (Figure 5B). Melatonin treatment was ineffective against pin- and pin + Aβ alterations of SOD activity in the hippocampus (*p* > 0.05).

#### 2.2.5. MDA Level in the FC

**Figure 6:**
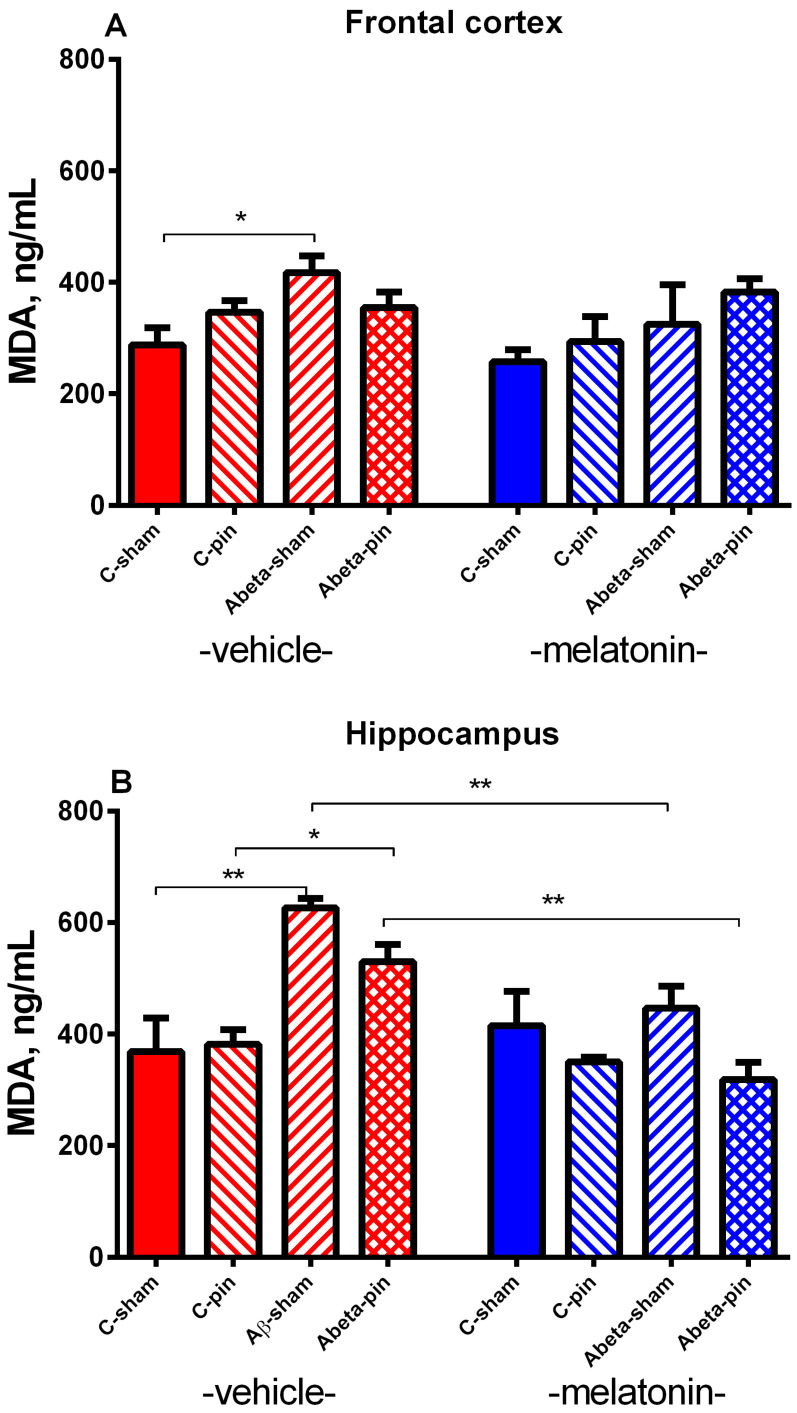
The influence of pinealectomy (pin) and chronic treatment with melatonin (mel) on MDA level in the frontal cortex (**A**) and hippocampus (**B**). Data are presented as mean ± S.E.M. * *p* = 0.021 Aβ-sham-veh group compared to C-sham-veh group (**A**) ** *p* = 0.002 Aβ-sham-veh group compared to C-sham-veh group; * *p* = 0.048 Aβ-pin-veh group compared to C-pin-veh group; ** *p* = 0.0039 Aβ-sham-mel group compared to Aβ-sham-veh group; ** *p* = 0.002, Aβ-pin-mel group compared to Aβ-pin-veh group (**B**).

Three-way ANOVA demonstrated a main effect of Aβ for the MDA levels in the FC [F_1,63_ = 3.466, *p* = 0.047]. The icv infusion of Aβ induced a significant elevation of the lipid peroxidation in the veh-treated rats (*p* = 0.021 Aβ-sham-veh group compared to C-sham-veh group) (Figure 6A). The chronic melatonin treatment could not prevent Aβ-pin induced elevation of the MDA level in the FC (*p* > 0.05).

#### 2.2.6. MDA Level in the Hippocampus

The analysis of variance showed a main effect of Aβ [F_1,63_ = 4.415, *p* = 0.044] as well as Pinealectomy × Aβ × Drug interaction [F_2,63_ = 6.090, *p* = 0.019] for the MDA levels in the hippocampus. Pinealectomy *per se* did not affect MDA levels (*p* > 0.05 C-pin-veh compared to the C-sham-veh group). The lipid peroxidation was significantly increased in the hippocampus after the icvAβ infusion in both the sham and pin group (*p* = 0.002 Aβ-sham-veh group compared to the C-sham-veh group; *p* = 0.048 Aβ-pin-veh group compared to C-pin-veh group) (Figure 6B). Melatonin was able to correct the increased MDA levels both in the Aβ-sham group (*p* = 0.0039 Aβ-sham-mel group Aβ-sham-veh group) and the Aβ-pin group (*p* = 0.002, Aβ-pin-mel group compared to Aβ-pin-veh group).

## 3. Discussion

The crucial role of OS in the pathogenesis of AD has been largely investigated, and numerous studies have reported that plasma levels of oxidative products are increased in patients with AD and can lead to neurodegeneration and impaired cognitive responses [44,45]. Melatonin has been reported to have multiple roles in the CNS, including chronotropic and antioxidant activity, improving neurogenesis and synaptic plasticity, neuroprotective, suppressing neuroinflammation, and enhancing memory function [46]. The underlying mechanism of the antioxidant activity of melatonin can be either as a direct free radical scavenger or by regulating the antioxidant or prooxidant enzymes via gene expression stimulation or increased activity of enzymes [47]. Although most of the published experiments point to melatonin as a free radical scavenger and antioxidant, there is some evidence to highlight the role of melatonin as a pro-oxidant mainly in cancer cells. [48]. The idea is that melatonin can increase oxidative stress and thus cause tumor cell death [48].

In the present study, we elaborated three rat models as follows: melatonin deficiency rat model induced by pinealectomy, icvAβ_1-42_ model of AD, and Aβ_1-42_ model of melatonin deficiency concomitant to AD pathology (pin+icvAβ_1-42_) to check whether the lack of melatonin in a condition of Aβ pathology could exacerbate model-related behavioral deficit and OS in the FC and hippocampus. In addition to verifying these rat models, we explored the potency of chronic melatonin treatment to correct the model-associated pathology. The present study’s findings suggest that while melatonin deficit exacerbates icvAβ-induced anxiety response and produces memory impairment in the pin+icvAβ_1-42_ model, chronic melatonin supplementation restores these behavioral deficits.

The enhanced anxiety resulting from pineal gland removal detected a month after the surgery procedure, was confirmed in two different tests-OF and EPM. However, our previous results and literature data demonstrated a divergence with a lack of effect on anxiety, anxiolysis, or anxiogenic response resulting from pinealectomy [49,50,51,52,53]. This suggests that the difference in the strain and the testing time after pinealectomy might be an important issue determining this behavioral response.

The detected icvAβ_1-42_-induced emotional disturbance associated with increased anxiety in rats was in line with our very recent study [46] as well as other reports [54].

The rats with melatonin deficiency concomitant to AD pathology also demonstrated impaired anxiety response which was more pronounced in the EPM than the icvAβ_1-42_-induced pathology. Moreover, melatonin supplementation successfully restored this behavioral deficit, preferably in the pin+icvAβ_1-42_ model (time in the aversive zone in the OF and EPM). Thus, we can speculate that model-related up-regulation of MT receptors in limbic structures including the amygdala and hippocampus determines the efficacy of melatonin treatment as was reported in patients with AD [26,27]. However, this presumption needs future investigation to confirm the role of MT receptors in the effect of melatonin supplementation on anxiety.

In support of the hypothesis that OS is elevated in the AD model [55,56,57,58], the present study confirmed previous reports which announced that the icvAβ_1-42_ infusion is associated with an elevated MDA level both in the FC and hippocampus. In turn, melatonin supplementation alleviated lipid oxidation.

However, the detected lipid peroxidation was not accompanied by significant adaptive changes of antioxidant markers GSH and SOD, respectively, as was reported by other authors in icvAβ_1-42_ mouse models [57]. Surprisingly, most of the previous reports considering the role of OS in Aβ pathology were conducted in mice while the few rat Aβ_1-42_ models did not explore changes of the antioxidant markers GSH and SOD.

On the other hand, although the model of melatonin deficiency in the present study did not affect the MDA level in the FC and hippocampus, it induced significant changes in the antioxidant system (SOD activity and GSH levels), suggesting that OS represents an essential part of brain pathology associated with pinealectomy. Several other studies have demonstrated that melatonin deficiency provokes OS in different rat tissues [59,60]. We confirmed a report by Tasdemir et al. [59] who show a decrease of GSH in the rat brain with pinealectomy, including in the FC and hippocampus. However, in contrast to Tasdemir et al. [59], who demonstrated decreased antioxidant markers in brain tissue, we found enhanced SOD activity in rats with pinealectomy. The discrepancy between our findings and those of Tasdemir et al. [59] is probably due to the difference in experimental procedures, brain structures examined, and measured time points of antioxidant markers after the surgery procedure. While the rats were decapitated a month after pinealectomy in our study, Tasdemir et al. [59] measured antioxidant markers six months later that might exacerbate the pathology associated with melatonin deficiency, including OS. In support of the presumption that the time window of pinealectomy might affect the expected pathology differently, our previous report shows that time of testing after pinealectomy is an important factor in determining responses [49]. The overproduction of SOD as a byproduct of oxygen metabolism plays a crucial role in the pathology of many diseases [61,62]. The idea that disturbed balance in SOD activity is toxic is associated with its main product H_2_O_2_ that might also be elevated due to SOD over-reactivity [61].

The original finding of the present study is that concomitant to the melatonin deficit model icvAβ_1-42_ infusion (pin+icvAβ_1-42_) represents a more relevant model of AD to study its pathology associated with OS. We report that the combined pin+icvAβ_1-42_ model is characterized by brain region-specific overproduction of SOD activity (more profound than in pinealectomy *per se*), as well as decreased GSH and lipid peroxidation. Furthermore, the supplementation with melatonin turned down the MDA levels in the pin+icvAβ_1-42_ model most probably by a direct mechanism of neutralizing the free radicals [60] and not through activation of the antioxidant system. The last assumption needs further clarification in future studies.

## 4. Materials and Methods

### 4.1. Animals

Ten weeks old adult male Sprague-Dawley rats (Charles River Lab., Wilmington, MA, USA), obtained from the vivarium of the Institute of Neurobiology, BAS, were kept in standard cages in groups of *n* = 3–4 under an artificial 12-h light/dark cycle, the temperature at 22–23 °C, and relative humidity 45%. Except during behavioral tests, the standard laboratory chow and water were at disposal *ad libitum*.

### 4.2. Experimental Design and Treatment with Melatonin

**Figure 7:**
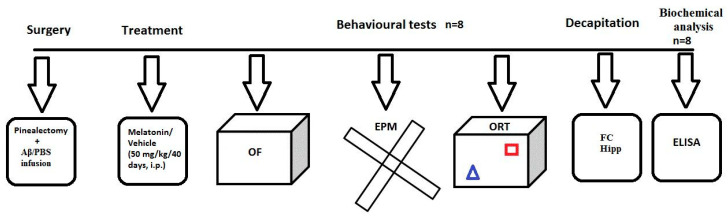
Schematic illustration of the experimental protocol. Groups and the number of animals used are shown. The number of animals for the behavioral test and biochemical analysis is given as the total number per group for each protocol. The brain structures used to determine oxidative markers were taken from the cohort of rats explored in the behavioral tests, open field, elevated plus-maze (EPM), and object recognition test (ORT).

Experimental design is presented in detail in Figure 7. In brief, the treatment with melatonin, dissolved in 1% hydroxyethylcellulose, started at the same day after the icv infusion of Aβ_1-42_, at a dose of 50 mg/kg, intraperitoneally (i.p.) and was injected about two hours before the onset of the dark phase for 40 days. The following eight groups were used: sham-operated and infused with PBS rats treated with vehicle (C-sham-veh group) (*n* = 8); rats with pinealectomy, infused with PBS and treated with vehicle (C-pin-veh) (*n* = 8), sham-operated, Aβ_1-42_-infused rats and treated with vehicle (Aβ-sham-veh) (*n* = 8); pin-operated, Aβ-infused and treated with vehicle (Aβ-pin-veh) (*n* = 8), sham-operated and infused with PBS rats treated with melatonin (C-sham-mel group) (*n* = 8); rats with pinealectomy, infused with PBS and treated with melatonin (C-pin-mel) (*n* = 8), sham-operated, Aβ_1-42_-infused rats and treated with melatonin (Aβ-sham-mel) (*n* = 8); pin-operated, Aβ-infused and treated with melatonin (Aβ-pin-mel) (*n* = 8).

### 4.3. Surgery and icv Injection of Aβ_1-42_

The rats under deep anesthesia induced with ketamine (80 mg/kg, i.p.) and xylazine (20 mg/kg, s.c.) were inserted on a stereotaxic apparatus (Narishige Sci. Inst. Labs, Tokyo, Japan). First, the pineal gland was rapidly removed through thin forceps according to the procedure described previously [49,63] and according to the procedure described by Hoffmann and Reiter [64]. We verified pinealectomy as a relevant model of melatonin deficit with a lack of melatonin in plasma during the dark phase [63]. After removal of the pineal gland, two cannulas were implanted bilaterally according to the atlas of Paxinos and Watson [65] at the following coordinates: (AP = −0.8, L = ± 1.5, H = 3.8). Amyloid β_1-42_ (100 µg; FOT Ltd., Sofia, Bulgaria) was dissolved in 100 μL PBS (vehicle solution) and incubated at room temperature for a week before using it to produce neurotoxic fibrils [44]. The Aβ_1-42_ intracerebroventricular (icv) infusion was performed with a 5-μL Hamilton microsyringe at a rate of 1 μL/min for 5 min. The microsyringe was left in place for an additional 2 min before removal. Matched procedures were carried out on the sham-operated group where PBS was infused. Several days after surgery the rats were injected with lactated Ringer’s and an antibiotic (gentamicin, s.c.).

### 4.4. Behavioral Tests

The behavioral tests were carried out between 10:00 a.m. and 12:00 p.m. in a separate soundproof room, under artificially diffused light, where the rats were accommodated for 30 min before the test. Also, the rats were repeatedly handled for a week before behavioral procedures to attenuate the effect of stress. For the open field (OF) a video tracking system (SMART PanLab software, Harvard Apparatus, USA) was used to analyze the collected data automatically. For the object recognition test (ORT) a video-recording system connected with DVR was used and the files were examined offline by two experienced observers.

*Open field test* was carried out as described previously [45,62]. In brief, each rat was placed in the central zone of the apparatus (100 × 100 cm × 60 cm) for a 5-min period. The following parameters were analyzed: the total distance traveled (cm), the vertical activity (number of rears), and the time spent in the center (cm). The apparatus was cleaned with 70% alcohol solution after each test.

*Elevated plus maze test* was performed as depicted in our previous reports [45,62]. The apparatus consisted of two open wooden arms (50 × 10 cm), two enclosed arms (50 × 10 × 50 cm), and a central platform (10 × 10 cm) elevated 50 cm above the floor. The tested animal was placed on the central square of the maze facing an open arm. The test was carried out for 5 min and the following parameters were calculated: (1) total locomotor activity (cm); (2) number of entries in the open arms; (3) time (sec) spent in the open arms. The apparatus was cleaned with 70% alcohol solution after each test.

*Object recognition test* was executed in an open plastic box (50 cm × 50 cm × 50 cm), with grey walls. The test consisted of three sessions. The flow, walls, and objects were cleaned after each trial with 70% alcohol solution. In the first session, each rat was placed in the box for 10 min without objects (accommodation). Twenty-four hours later, the second session was performed, and the rat was exposed to identical objects made of glass (A1 and A2) for 3 min. The objects were positioned about 10 cm away from the opposite corners. One hour later, one of the objects was replaced with a new one made from plastic (the novel object B), and test the third session was carried out again for 3 min. The number (count) and time (sec) of exploration defined as the nose approach to the object in the third session were recorded. The discrimination index DI (count and time) of exploration for each object was calculated as follows: B/B + A.

### 4.5. Detection of Markers Related to Oxidative Stress in the Homogenates from the Frontal Cortex and Hippocampus

After behavioral tests, all rats were decapitated with a guillotine after mild anesthesia with CO_2_ and the whole brains were removed. FC and hippocampi were carefully excised. Each of the frontal cortex and hippocampal samples were weighted and homogenized (1:10) with SONOPULS ultrasonic homogenizer (BANDELIN electronic GmbH&Co. KG, Berlin) in ice-cold 0.9% saline (6 mice/group). The homogenates were centrifuged at 3000× *g* for 10 min at 4 °C to obtain the supernatants. The supernatants were diluted with the appropriate buffer and were used for assays of GSH and MDA levels and SOD-specific activities.

#### 4.5.1. Determination of GSH

Quantitative analysis of GSH levels was performed with Rat Glutathione (GSH) ELISA kit (Cat. No MBS774706, MyBioSource, Inc., San Diego, CA 92195-3308 USA) according to the manufacturer’s instructions. The absorbance was measured using a microplate reader (Tecan Infinite F200 PRO (Tecan Austria GmbH, Salzburg, Austria) at a wavelength of 450 nm.

#### 4.5.2. Determination of SOD

Quantitative analysis of SOD levels was performed with Rat Super Oxide Dismutase ELISA kit (Cat. No MBS162314, MyBioSource, Inc., San Diego, CA 92195-3308 USA) according to the manufacturer’s instructions. The absorbance was measured using a microplate reader (Tecan Infinite F200 PRO (Tecan Austria GmbH, Salzburg, Austria) at a wavelength of 450 nm.

#### 4.5.3. Determination of MDA

Quantitative analysis of malondialdehyde (MDA) levels was performed with Rat Malondialdehyde ELISA kit (Cat. No MBS738685, MyBioSource, Inc., San Diego, CA 92195-3308 USA) according to the manufacturer’s instructions. The absorbance was measured using a microplate reader (Tecan Infinite F200 PRO (Tecan Austria GmbH, Salzburg, Austria) at a wavelength of 450 nm.

#### 4.5.4. Statistical Analysis

Three-way ANOVA analyzed the behavioral and biochemical data were by with factors: Pinealectomy (sham and pin), Aβ (control, Aβ), and Drug (vehicle, melatonin) followed by *post hoc* Bonferroni test in case of detected significant difference (SigmaStat 11.0). When data were not homogenously distributed, nonparametric tests were applied (Kruskal–Wallis on ranks followed by the Mann–Whitney U test). The significant level was set at *p* ≤ 0.05.

## 5. Conclusions

In the present study, we introduced a new pin+icvAβ_1-42_ rat model of AD with concomitant melatonin deficiency and compared its behavioral and OS pathology with the pin model and icvAβ_1-42_ model, respectively. Our results suggest that the pin+icvAβ_1-42_ rat model induces more pronounced anxiety and OS. The latter is better expressed in the hippocampus. Melatonin treatment minimizes these neurodegenerative features, and OS elevation most probably by a direct free radical scavenger mechanism.

## References

[1] Jiang T., Sun Q., Chen S. (2016). Oxidative stress: A major pathogenesis and potential therapeutic target of antioxidative agents in Parkinson’s disease and Alzheimer’s disease. Prog. Neurobiol..

[2] Markesbery W.R., Lovell M.A. (2006). DNA oxidation in Alzheimer’s disease. Antioxid. Redox Signal..

[3] Markesbery W.R., Lovell M.A. (2007). Damage to lipids, proteins, DNA, and RNA in mild cognitive impairment. Arch. Neurol..

[4] Moreira P.I., Nunomura A., Nakamura M., Takeda A., Shenk J.C., Aliev G., Smith M.A., Perry G. (2008). Nucleic acid oxidation in Alzheimer disease. Free Radic. Biol. Med..

[5] Oddo S., Caccamo A., Shepherd J.D., Murphy M.P., Golde T.E., Kayed R., Metherate R., Mattson M.P., Akbari Y., LaFerla F.M. (2003). Triple-transgenic model of Alzheimer’s disease with plaques and tangles: Intracellular Abeta and synaptic dysfunction. Neuron.

[6] Halliwell B. (1992). Reactive oxygen species and the central nervous system. J. Neurochem..

[7] Halliwell B. (2001). Role of free radicals in the neurodegenerative diseases: Therapeutic implications for antioxidant treatment. Drugs Aging.

[8] Faraci F.M. (2006). Reactive oxygen species: Influence on cerebral vascular tone. J. Appl. Physiol..

[9] Mantha A.K., Moorthy K., Cowsik S.M., Baquer N.Z. (2006). Neuroprotective role of neurokinin B (NKB) on beta-amyloid (25–35) induced toxicity in aging rat brain synaptosomes: Involvement in oxidative stress and excitotoxicity. Biogerontology.

[10] Jang Y.C., Rodriguez K., Lustgarten M.S., Muller F.L., Bhattacharya A., Pierce A., Choi J.J., Lee N.H., Chaudhuri A., Richardson A.G. (2020). Superoxide-mediated oxidative stress accelerates skeletal muscle atrophy by synchronous activation of proteolytic systems. GeroScience.

[11] Jang Y.C., Lustgarten M.S., Liu Y., Muller F.L., Bhattacharya A., Liang H., Salmon A.B., Brooks S.V., Larkin L., Hayworth C.R. (2010). Increased superoxide in vivo accelerates age-associated muscle atrophy through mitochondrial dysfunction and neuromuscular junction degeneration. FASEB J..

[12] Praticò D., Delanty N. (2000). Oxidative injury in diseases of the central nervous system: Focus on Alzheimer’s disease. Am. J. Med..

[13] Floyd R.A. (1999). Antioxidants, oxidative stress, and degenerative neurological disorders. Proc. Soc. Exp. Biol. Med..

[14] Cetin F., Kishore U. (2013). Role of oxidative stress in Aβ animal model of Alzheimer’s disease: Vicious circle of apoptosis, nitric oxide and age. Neurodegenerative Diseases.

[15] Engelhart M.J., Geerlings M.I., Ruitenberg A., van Swieten J.C., Hofman A., Witteman J.C., Breteler M.M. (2002). Dietary intake of antioxidants and risk of Alzheimer disease. JAMA.

[16] Morris M.C., Evans D.A., Bienias J.L., Tangney C.C., Bennett D.A., Aggarwal N., Wilson R.S., Scherr P.A. (2002). Dietary intake of antioxidant nutrients and the risk of incident Alzheimer disease in a biracial community study. JAMA.

[17] Luchsinger J.A., Tang M.X., Shea S., Mayeux R. (2003). Antioxidant vitamin intake and risk of Alzheimer disease. Arch. Neurol..

[18] Bubenik G.A. (2002). Gastrointestinal melatonin: Localization, function, and clinical relevance. Dig. Dis. Sci..

[19] Reiter R.J., Tan D.X., Osuna C., Gitto E. (2000). Actions of melatonin in the reduction of oxidative stress. A review. J. Biomed. Sci..

[20] Bondy S.C., Sharman E.H. (2007). Melatonin and the aging brain. Neurochem. Int..

[21] Morabito R., Remigante A., Marino A. (2019). Melatonin Protects Band 3 Protein in Human Erythrocytes against H_2_O_2_-Induced Oxidative Stress. Molecules.

[22] Lin L., Huang Q.X., Yang S.S., Chu J., Wang J.Z., Tian Q. (2013). Melatonin in Alzheimer’s disease. Int. J. Mol. Sci..

[23] Ohashi Y., Okamoto N., Uchida K., Iyo M., Mori N., Morita Y. (1999). Daily rhythm of serum melatonin levels and effect of light exposure in patients with dementia of the Alzheimer’s type. Biol. Psychiat..

[24] Wu Y.H., Feenstra M.G., Zhou J.N., Liu R.Y., Torano J.S., van Kan H.J., Fischer D.F., Ravid R., Swaab D.F. (2003). Molecular changes underlying reduced pineal melatonin levels in Alzheimer’s disease: Alterations in preclinical and clinical stages. J. Clin. Endocr. Metab..

[25] Zhou J.N., Liu R.Y., Kamphorst W., Hofman M.A., Swaab D.F. (2003). Early neuropathological Alzheimer’s changes in aged individuals are accompanied by decreased cerebrospinal fluid melatonin levels. J. Pineal Res..

[26] Savaskan E., Olivieri G., Meier F., Brydon L., Jockers R., Ravid R., Wirz-Justice A., Muller-Spahn F. (2002). Increased melatonin 1a-receptor immunoreactivity in the hippocampus of Alzheimer’s disease patients. J. Pineal Res..

[27] Savaskan E., Ayoub M.A., Ravid R., Angeloni D., Fraschini F., Meier F., Eckert A., Müller-Spahn F., Jockers R. (2005). Reduced hippocampal MT2 melatonin receptor expression in Alzheimer’s disease. J. Pineal Res..

[28] Alluri H., Wilson R.L., Anasooya Shaji C., Wiggins-Dohlvik K., Patel S., Liu Y., Peng X., Beeram M.R., Davis M.L., Huang J.H. (2016). Melatonin Preserves Blood-Brain Barrier Integrity and Permeability via Matrix Metalloproteinase-9 Inhibition. PLoS ONE.

[29] Namyen J., Permpoonputtana K., Nopparat C., Tocharus J., Tocharus C., Govitrapong P. (2020). Protective Effects of Melatonin on Methamphetamine-Induced Blood-Brain Barrier Dysfunction in Rat Model. Neurotox. Res..

[30] Brusco L.I., Márquez M., Cardinali D.P. (2000). Melatonin treatment stabilizes chronobiologic and cognitive symptoms in Alzheimer’s disease. Neuroendocrinol. Lett..

[31] Cardinali D.P., Brusco L.I., Liberczuk C., Furio A.M. (2002). The use of melatonin in Alzheimer’s disease. Neuroendocrinol. Lett..

[32] Singer C., Tractenberg R.E., Kaye J., Schafer K., Gamst A., Grundman M., Thomas R., Thal L.J., Alzheimer’s Disease Cooperative Study (2003). A multicenter, placebo-controlled trial of melatonin for sleep disturbance in Alzheimer’s disease. Sleep.

[33] O′Kelly C., Wang X., Raso J., Moreau M., Mahood J., Zhao J., Bagnall K. (1999). The production of scoliosis after pinealectomy in young chickens, rats, and hamsters. Spine.

[34] Beriwal N., Namgyal T., Sangay P., Al Quraan A.M. (2019). Role of immune-pineal axis in neurodegenerative diseases, unraveling novel hybrid dark hormone therapies. Heliyon.

[35] Cetin F., Dincer S. (2007). The effect of intrahippocampal beta amyloid (1-42) peptide injection on oxidant and antioxidant status in rat brain. Ann. N. Y. Acad. Sci..

[36] Cioanca O., Hritcu L., Mihasan M., Hancianu M. (2013). Cognitive-enhancing and antioxidant activities of inhaled coriander volatile oil in amyloid β(1-42) rat model of Alzheimer’s disease. Physiol. Behav..

[37] Olariu A., Tran M.H., Yamada K., Mizuno M., Hefco V., Nabeshima T. (2001). Memory deficits and increased emotionality induced by beta-amyloid (25-35) are correlated with the reduced acetylcholine release and altered phorbol dibutyrate binding in the hippocampus. J. Neural. Transm..

[38] Ruan L., Kong Y., Wang J.M., Le Y. (2010). Chemoattractants and receptors in Alzheimer’s disease. Front. Biosci..

[39] Butterfield D.A., Swomley A.M., Sultana R. (2013). Amyloid *β*-Peptide (1-42)-Induced Oxidative Stress in Alzheimer Disease: Importance in Disease Pathogenesis and Progression. Antioxid. Redox Signal..

[40] Wang C., Yang X.M., Zhuo Y.Y., Zhou H., Lin H.B., Cheng Y.F., Xu J.P., Zhang H.T. (2012). The phosphodiesterase-4 inhibitor rolipram reverses Aβ-induced cognitive impairment and neuroinflammatory and apoptotic responses in rats. Int. J. Neuropsychopharmacol..

[41] Jhoo J.H., Kim H.C., Nabeshima T., Yamada K., Shin E.J., Jhoo W.K., Kim W., Kang K.S., Jo S.A., Woo J.I. (2004). Beta-amyloid (1-42)-induced learning and memory deficits in mice: Involvement of oxidative burdens in the hippocampus and cerebral cortex. Behav. Brain Res..

[42] Srivareerat M., Tran T.T., Salim S., Aleisa A.M., Alkadhi K.A. (2011). Chronic nicotine restores normal Aβ levels and prevents short-term memory and E-LTP impairment in Aβ rat model of Alzheimer’s disease. Neurobiol. Aging.

[43] Eftekharzadeh B., Ramin M., Khodagholi F., Moradi S., Tabrizian K., Sharif R., Azami K., Beyer C., Sharifzadeh M. (2012). Inhibition of PKA attenuates memory deficits induced by β-amyloid (1-42), and decreases oxidative stress and NF-κB transcription factors. Behav. Brain Res..

[44] Asadbegi M., Yaghmaei P., Salehi I., Ebrahim-Habibi A., Komaki A. (2016). Neuroprotective effects of metformin against Aβ-mediated inhibition of long-term potentiation in rats fed a high-fat diet. Brain Res. Bull..

[45] Tchekalarova J., Atanasova D., Nenchovska Z., Atanasova M., Kortenska L., Gesheva R., Lazarov N. (2017). Agomelatine protects against neuronal damage without preventing epileptogenesis in the kainate model of temporal lobe epilepsy. Neurobiol. Dis..

[46] Ilieva K., Atanasova M., Atanasova D., Kortenska L., Tchekalarova J. (2021). Chronic agomelatine treatment alleviates icvAβ-induced anxiety and depressive-like behavior through affecting Aβ metabolism in the hippocampus in a rat model of Alzheimer’s disease. Physiol. Behav..

[47] Schipper H.M., Chertkow H., Mehindate K., Frankel D., Melmed C., Bergman H. (2000). Evaluation of heme oxygenase-1 as a systemic biological marker of sporadic AD. Neurology.

[48] Zhang H.M., Zhang Y. (2014). Melatonin: A well-documented antioxidant with conditional pro-oxidant actions. J. Pineal Res..

[49] Tchekalarova J., Nenchovska Z., Atanasova D., Atanasova M., Kortenska L., Stefanova M., Alova L., Lazarov N. (2016). Consequences of long-term treatment with agomelatine on depressive-like behavior and neurobiological abnormalities in pinealectomized rats. Behav. Brain Res..

[50] Appenrodt E., Schwarzberg H. (2000). Central vasopressin administration failed to influence anxiety behavior after pinealectomy in rats. Physiol. Behav..

[51] Bustamante-García R., Lira-Rocha A.S., Espejo-González O., Gómez-Martínez A.E., Picazo O. (2014). Anxiolytic-like effects of a new 1-N substituted analog of melatonin in pinealectomized rats. Prog. Neuro-Psychopharmacol. Biol. Psychiatry.

[52] Karakaş A., Coşkun H., Kaya A., Kücük A., Gündüz B. (2011). The effects of the intraamygdalar melatonin injections on the anxiety like behavior and the spatial memory performance in male Wistar rats. Behav. Brain Res..

[53] Zahra E., Siham O., Abdelhalim M., Aboubakr E., Ali O. (2012). Pinealectomy and exogenous melatonin regulate anxiety-like and depressive-like behaviors inmale and female wistar rats. Neurosci. Med..

[54] Sharma S., Verma S., Kapoor M., Saini A., Nehru B. (2016). Alzheimer’s disease like pathology induced six weeks after aggregated amyloid-beta injection in rats: Increased oxidative stress and impaired long-term memory with anxiety-like behavior. Neurol. Res..

[55] Boyd-Kimball D., Sultana R., Mohmmad-Abdul H., Butterfield D.A. (2004). Rodent Abeta(1-42) exhibits oxidative stress properties similar to those of human Abeta(1-42): Implications for proposed mechanisms of toxicity. J. Alzheimer’s Dis..

[56] Li J., Wang C., Zhang J.H., Cai J.M., Cao Y.P., Sun X.J. (2010). Hydrogen-rich saline improves memory function in a rat model of amyloid-beta-induced Alzheimer’s disease by reduction of oxidative stress. Brain Res..

[57] Saeed K., Shah S.A., Ullah R., Alam S.I., Park J.S., Saleem S., Jo M.H., Kim M.W., Hahm J.R., Kim M.O. (2020). Quinovic Acid Impedes Cholesterol Dyshomeostasis, Oxidative Stress, and Neurodegeneration in an Amyloid-β-Induced Mouse Model. Oxidative Med. Cell. Longev..

[58] Ahmad S., Orellana A., Kohler I., Frölich L., de Rojas I., Gil S., Boada M., Hernández I., Hausner L., Bakker M.H.M. (2020). Association of lysophosphatidic acids with cerebrospinal fluid biomarkers and progression to Alzheimer’s disease. Alzheimer’s Res. Ther..

[59] Tasdemir S., Samdanci E., Parlakpinar H., Polat A., Tasdemir C., Cengiz N., Sapmaz H., Acet A. (2012). Effects of pinealectomy and exogenous melatonin on the brains, testes, duodena and stomachs of rats. Eur. Rev. Med. Pharmacol. Sci..

[60] Sahna E., Parlakpinar H., Vardi N., Ciğremis Y., Acet A. (2004). Efficacy of melatonin as protectant against oxidative stress and structural changes in liver tissue in pinealectomized rats. Acta Histochem..

[61] McCord J.M. (2000). The evolution of free radicals and oxidative stress. Am. J. Med..

[62] McCord J.M. (2008). Superoxide dismutase, lipid peroxidation, and bell-shaped dose response curves. Dose-Response.

[63] Tchekalarova J., Stoyanova T., Nenchovska Z., Ivanova N., Atanasova D., Atanasova M., Georgieva K. (2020). Effect of endurance training on diurnal rhythms of superoxide dismutase activity, glutathione and lipid peroxidation in plasma of pinealectomized rats. Neurosci. Lett..

[64] Hoffman R.A., Reiter R.J. (1965). Rapid pinealectomy in hamsters and other small rodents. Anat. Rec..

[65] Paxinos G., Watson C. (2007). The Rat Brain in Stereotaxic Coordinates.

